# Supplementary material for: Distinct contributions of prefrontal, parietal, and cingulate signals to exploratory decisions
Source: Commun Biol. 2026 Jan 22;9:272. doi: 10.1038/s42003-026-09550-w (PMC12916896; doi:10.1038/s42003-026-09550-w)
Supplement: Supplementary file 2 — Supplementary information [file 42003_2026_9550_MOESM2_ESM.pdf]

Distinct contributions of prefrontal, parietal, and cingulate signals to exploratory decisions.

Victor K. S. Chan, Nicole H. L. Wong, Tsz-Fung Woo, Kei Watanabe, Masahiko Haruno, Chun-Kit Law, Bolton K. H. Chau

Supplementary Information

Supplementary Figures

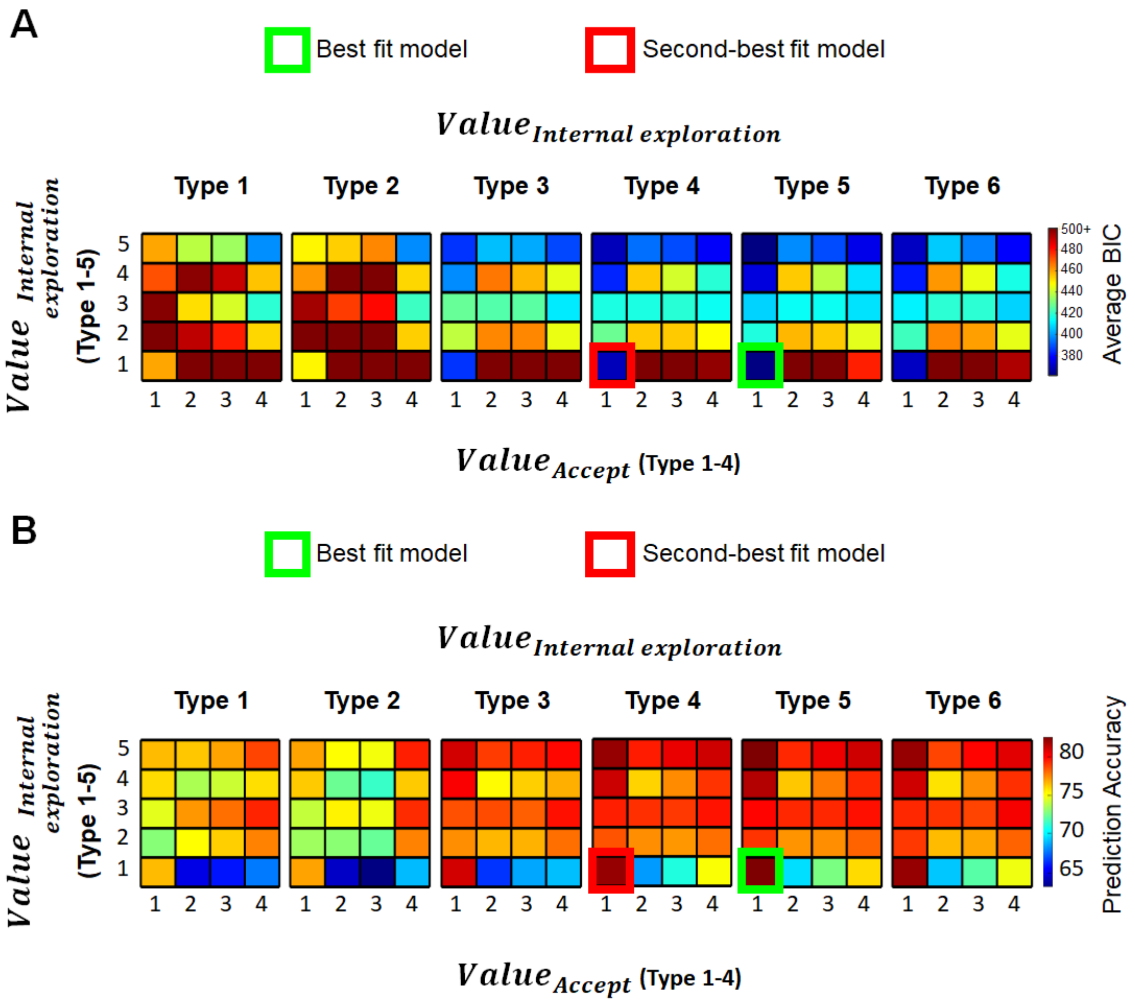

Supplementary Figure S1. A total of 120 models were generated. This was based on combining six types of internal exploration value ($Value_{Internal\ exploration}$), five types of external exploration value ($Value_{External\ exploration}$), and four types of accept value ($Value_{Accept}$). Details of each value function is described below. **(a)** The Bayesian information criterion (BIC) values of each model was estimated based applying a general linear model on each participant's data and then averaged across participants (see Methods). The results suggested that the best fit model with the lowest average BIC involves internal exploration

value type 5, external exploration value type 1, and accept value type 1 (green box; average BIC = 361.26). We noticed that an alternative model that involves internal exploration value type 5, external exploration value type 5, accept value type 1 was equally good because their variance explained are identical, but we selected the former model because it is involves more parsimonious operational definition. We noticed that the second-best fit model involves internal exploration value type 4, external exploration value type 1, and accept value type 1 (mean BIC = 369.37). In Supplementary Fig. S6 and Supplementary Fig. S7, we showed that applying this alternative model provides comparable results in our subsequent behavioural and neural data analyses. **(b)** The accuracy of each model's prediction of participants' choices**.**

The six internal exploration value types were formulated as follows:

$$Value_{Internal\ exploration(1)} = maxVariance_{\ (option)}$$

$$Value_{Internal\ exploration(2)} = maxStandardDeviation_{\ (option)}$$

$$Value_{Internal\ exploration(3)} = max(maxPoint_{\ (option)} - minPoint_{\ (option)})$$

$$Value_{Internal\ exploration(4)} = max(Point_{\ (option)} * Variance_{\ (option)})$$

$$Value_{Internal\ exploration(5)} = max(Point_{\ (option)} * StandardDeviation_{\ (option)})$$

$$Value_{Internal\ exploration(6)} = max\ (Point_{(option)} * (maxPoint_{(option)} - minPoint_{(option)}))$$

The first to the third type of internal exploration value were defined as the greatest variance ($Value_{Internal\ exploration(1)}$), the greatest standard deviation ($Value_{Internal\ exploration(2)}$) and the greatest point range ($Value_{Internal\ exploration(3)}$) amongst all revealed options respectively. The fourth to the sixth type of internal exploration value were defined as the greatest product between the point and the variance ($Value_{Internal\ exploration(4)}$), the greatest product between the point and the standard deviation ($Value_{Internal\ exploration(5)}$), and the greatest product of the point and the range of the value ($Value_{Internal\ exploration(6)}$) amongst all revealed options respectively.

The five types of the external exploration values were formulated as follows:

$$Value_{External\ exploration(1)} = \overline{Point}_{\ (all\ options)}$$

$$Value_{External\ exploration(2)} = \overline{Point}_{(all\ revealed\ options)}$$

$$Value_{External\ exploration(3)} = Point_{(new\ unveiled\ option)}$$

$$Value_{External\ exploration(4)} = \overline{Point}_{(all\ options)} - \overline{Point}_{(all\ revealed\ options)}$$

$$Value_{External\ exploration(5)} = \overline{Point}_{(all\ options)} - Point_{(the\ best\ option)}$$

The first type of the external exploration value was formulated as the average point of all hidden and revealed options ($Value_{External\ exploration(1)}$). The second type of the external exploration value was formulated as the average point amongst all revealed options ($Value_{External\ exploration\ (2)}$), assuming a smaller average point would drive the external exploration for better alternatives.

Previous findings about external exploration suggested that it is an adaptive behaviour in response to a new environment [1–3]. The third type was then formulated as the point of the new option ($Value_{External\ exploration(3)}$). The new option could be the most recently revealed or internal explored option.

The fourth and fifth types addressed whether the point difference between revealed options and the environment might lead to an external exploration decision. They were formulated as the average point of all hidden and revealed options minus the average point of all revealed options ($Value_{External\ exploration(4)}$), and the average point of all options minus the average point of the best option ($Value_{External\ exploration(5)}$).

The three accept value types were formulated as follows:

$$Value_{Accept(1)} = Point_{(the\ best\ option)}$$

$$Value_{Accept(2)} = \frac{Point_{(the\ best\ option)}}{Variance_{(the\ best\ option)}}$$

$$Value_{Accept(3)} = \frac{Point_{(the\ best\ option)}}{StandardDeviation_{(the\ best\ option)}}$$

$$Value_{Accept(4)} = Point_{(the\ best\ option)} - Variance_{(the\ best\ option)}$$

Considering that participants might accept the option with the greatest average point, the first type of accept value ($Value_{Accept(1)}$) was defined as the average point of the best option. In order to investigate whether uncertainty of the best option would discount the value or not, the variance ($Value_{Accept\ (2)}$) and standard deviation ($Value_{Accept\ (3)}$) was incorporated in the equation for Type 2 and 3 respectively. As the mean-variance trade-off is known to drive

decision making. The fourth type of accept value ($Value_{Accept\ (4)}$) was defined as the comparison between the average point and the variance of the best option.

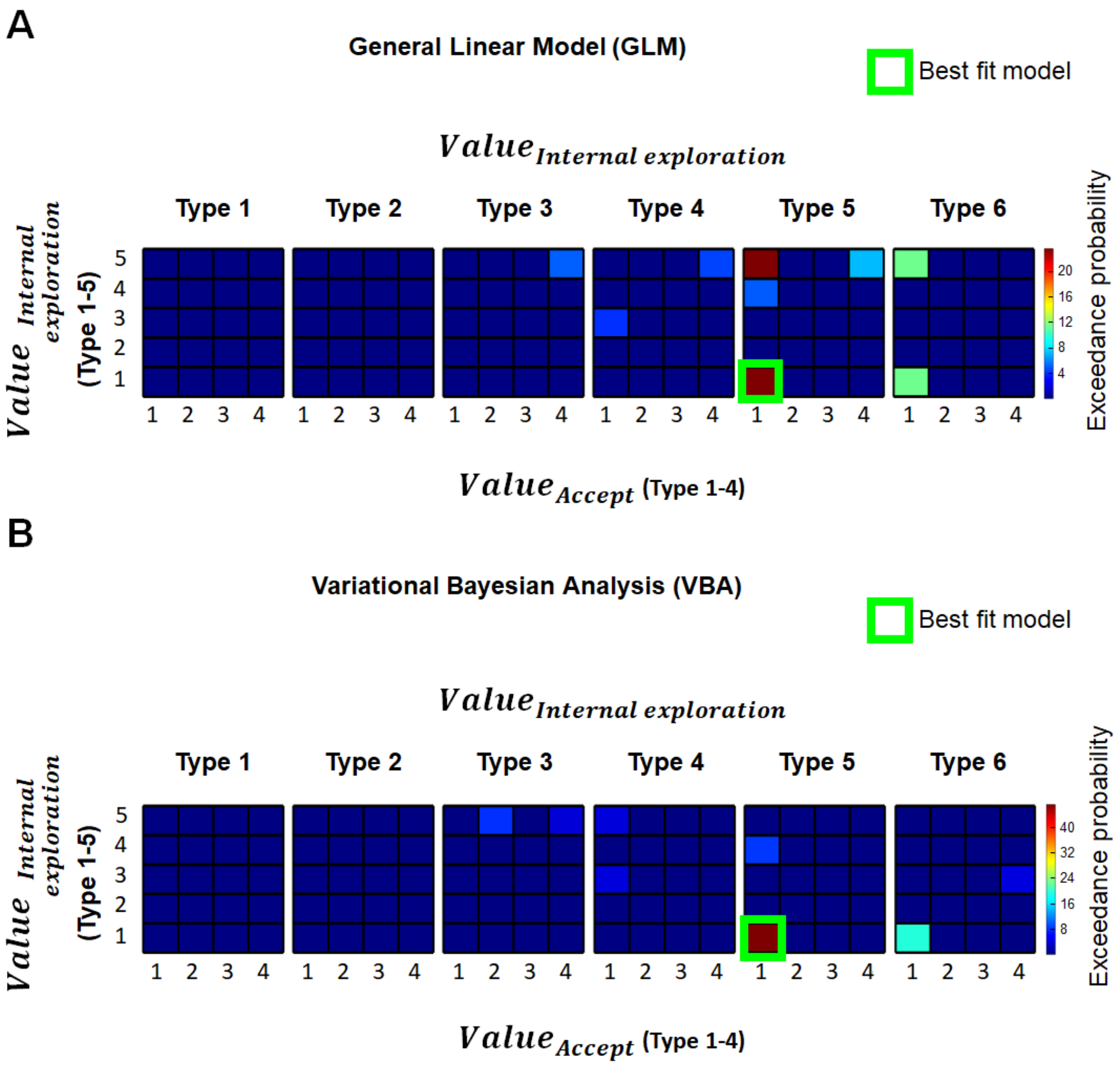

Supplementary Figure S2. Bayesian model selection results. (a) General Linear Model**. (b)** Variational Bayesian Analysis (VBA)**.** Exceedance probabilities for each candidate model, representing the probability that a given model is more frequent than all other models in the population. The same model showed the highest exceedance probability (the Best-fit model). This analysis corroborated our initial findings, with the same model emerging as the best-fit, thus strengthening our conclusions through converging evidence from multiple analytical approaches. Note that the model with type 1 external exploration value, type 5 internal exploration value and type 1 accept value (EEV1, IEV5, ACCV1) and the model (EEV5, IEV5, ACCV1) are statistically equivalent under the GLM because EEV5 $\equiv$ EEV1 − ACCV1 (Please see the operational definition from Supplementary Figure S1). In the VBA framework, a modest complexity penalty is applied to the redundant term in EEV5, resulting in a slightly lower mean log-likelihood (model using EEV5: −210.29 versus model using EEV1: −209.71). The more parsimonious specification therefore attains higher probability.

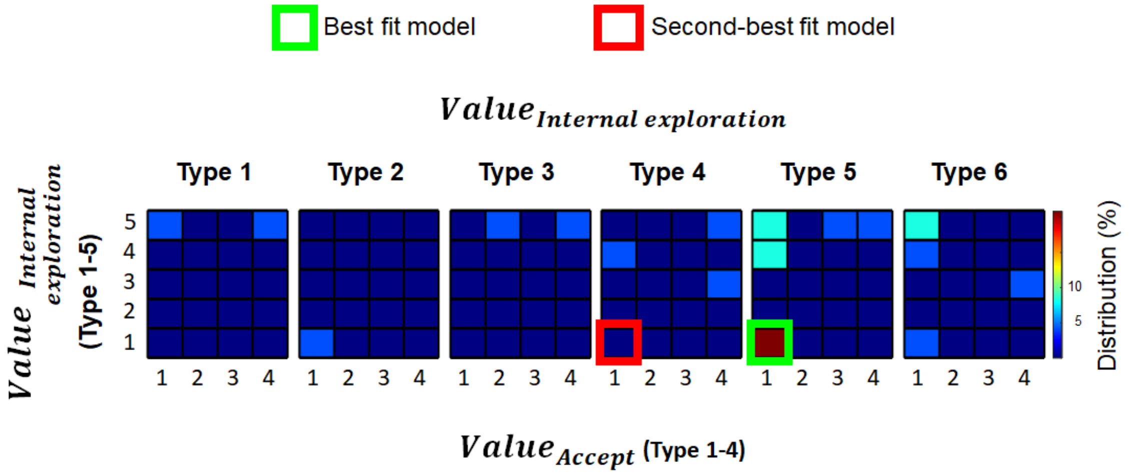

Supplementary Figure. S3. The distribution of best-fit model at participant level. In this analysis, we counted the percentage of participants that was best described by each model. The results showed that the best-fit model at the group level (labelled by a green box) was also the model that best describes the greatest proportion of participants' behaviour**.**

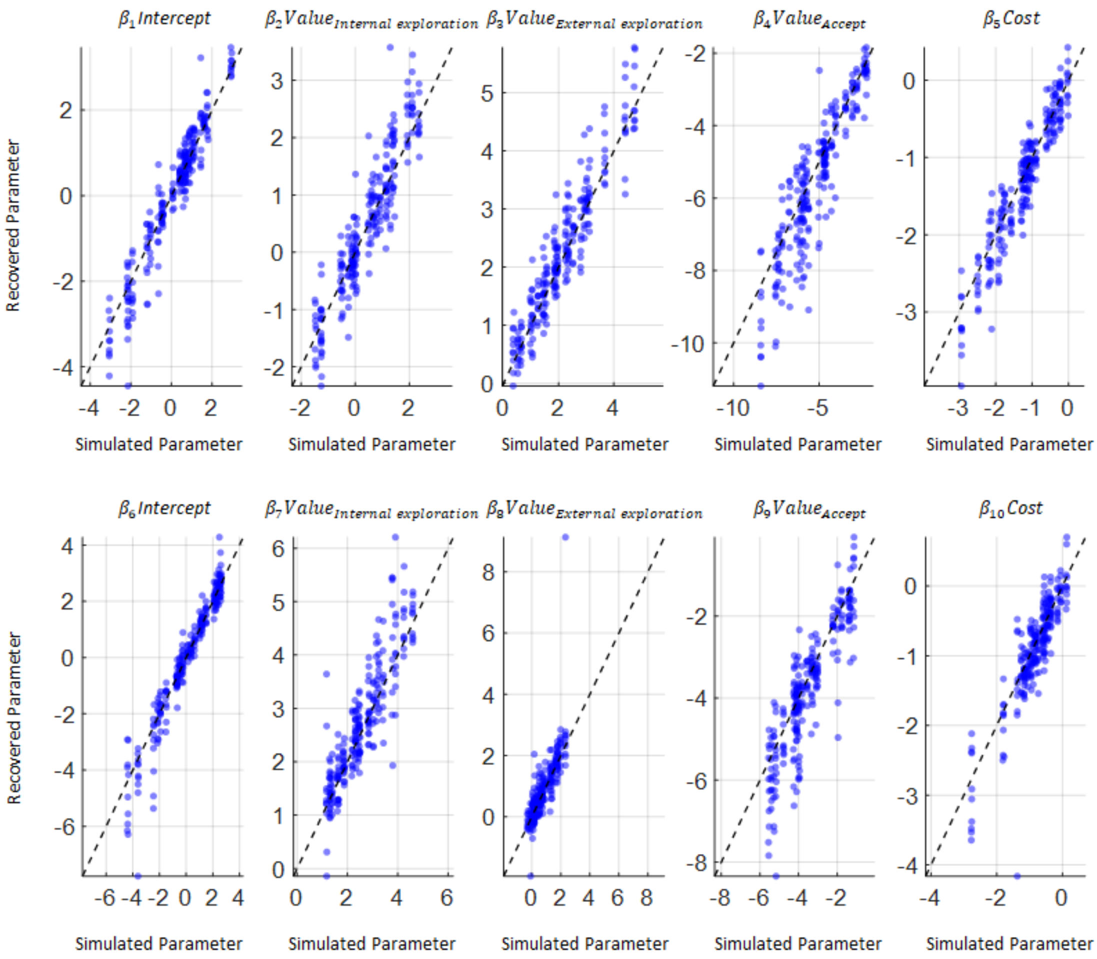

Supplementary Figure. S4. Parameter recovery analysis of best-fit model. The fitted parameters of each participant were first added with Gaussian noise ($\sigma = 0.01$ of the group SD), employed to generate simulated choice probability data, which was then randomly converted to simulated, categorical choice data. The simulation of each participants data was

iterated for 10 times, resulting in choice data from 260 simulated participants. These simulated data were then fitted using the same model to test the correlation between the true, simulated parameters and the fitted parameters. The results showed that the fitting procedures could successfully recover all ten parameters with high precision (all correlations: rs > 0.801). Black dashed lines indicate perfect recovery, and filled circles represents individual simulations.

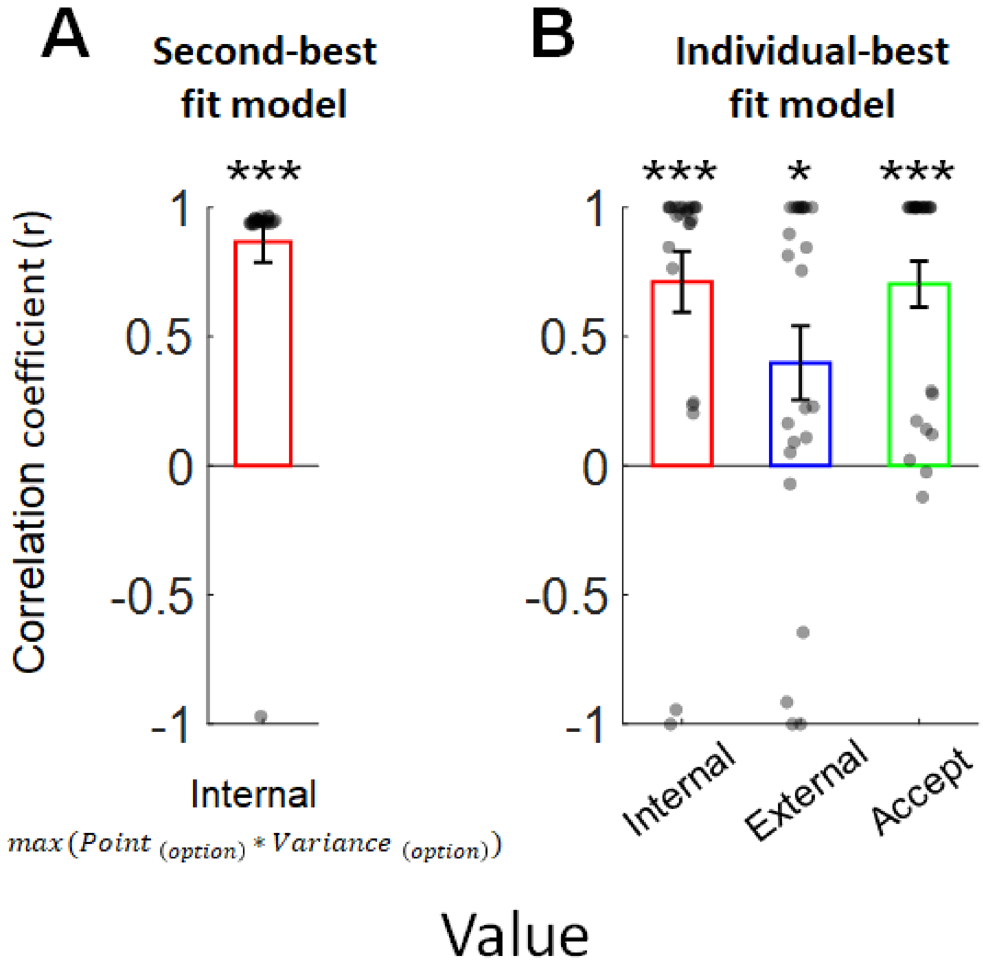

Supplementary Figure S5. Correlation between the value estimates of the best fit model and two alternative models (i.e., second-best fit model and individual participants' best fit model) (n = 24). **(a).** Given that the only difference between the best fit model and the second-best fit model was the operational definition of internal exploration value (Supplementary Fig. S1), we only estimated the internal exploration value from the second-best fit model and correlated it with the best fit model, the correlation was significant ($r = 0.868$, $p < 0.001$). **(b).** An extra model considered each individual participants' best fit model was also defined as another competitive model for model comparisons. The correlations between the estimated internal exploration value ($r = 0.712$, $p < 0.001$), the estimated external exploration value ($r = 0.398$, $p = 0.011$), the estimated accept value ($r = 0.703$, $p < 0.001$) of the best fit model and individual participants' best fit model were significant. *** denotes $p \leqslant 0.001$.

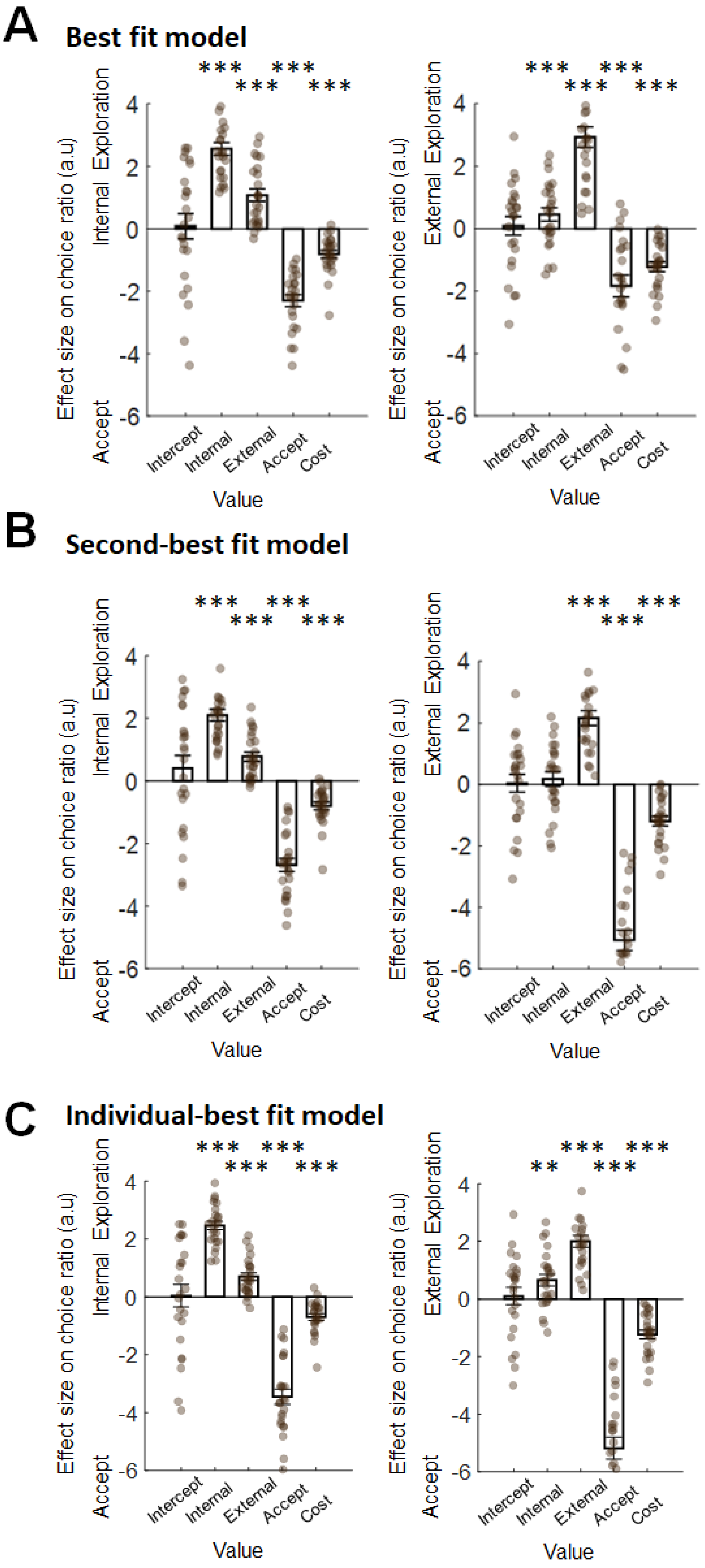
A
Best fit model
B
Second-best fit model
C
Individual-best fit model
Accept Effect size on choice ratio (a.u)
Internal Exploration
External Exploration
Intercept
Internal
External
Accept
Cost
Value
***
**

Supplementary Figure S6. Alternative models generated similar behavioural results (*n* = 24). **(a)** The results generated using the best fit model show in Fig. 5a is presented again. The analysis was repeated using the **(b)** second-best fit model and **(c)** individual participants' best fit model. These alternative models generated comparable results. * denotes $p < 0.05$, ** denotes $p < 0.01$, *** denotes $p \leq 0.001$. Error bars represent ± SEM.

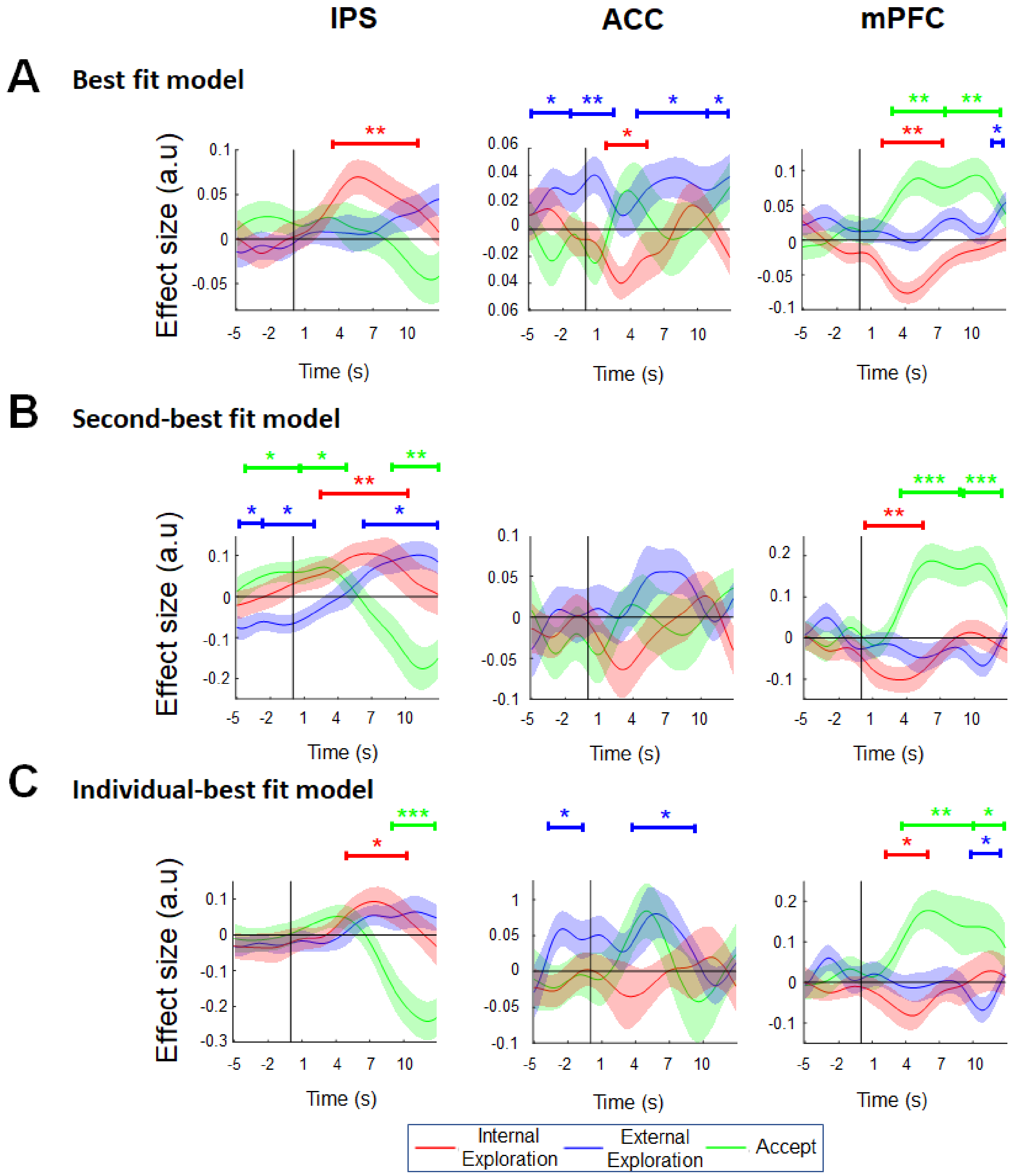

Supplementary Figure S7. Alternative models generated similar neural results. (a) The results generated using the best fit model show in Fig. 7b is presented again. The analysis was repeated using the **(b)** second-best fit model and **(c)** individual participants' best fit model. These alternative models generated comparable results. Time-locked to the stimulus onset (solid black line). * denotes $p < 0.05$, ** denotes $p < 0.01$, *** denotes $p \leq 0.001$. Shaded areas represent ± SEM.

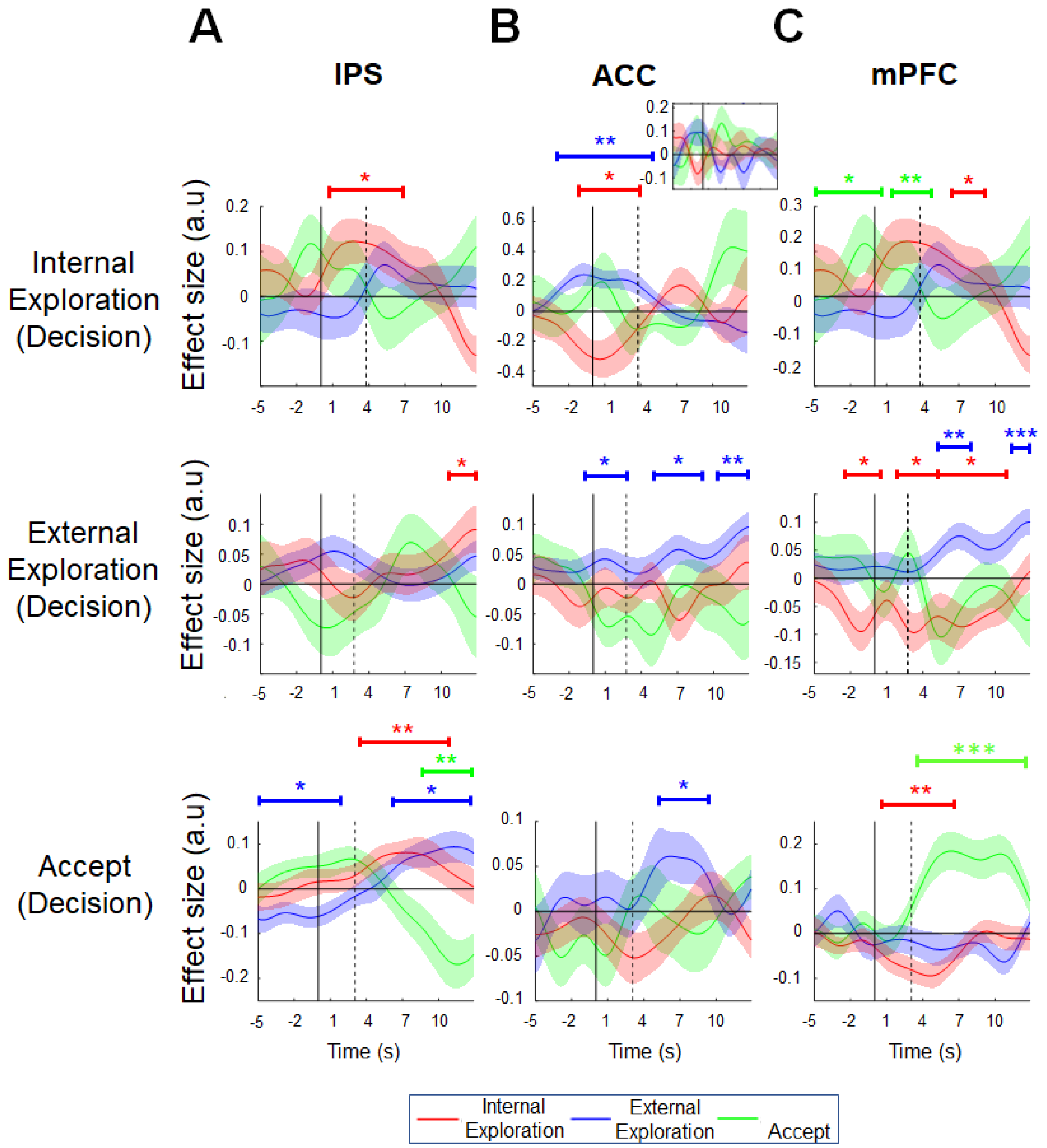

Supplementary Figure S8. Time courses of the internal exploration, external exploration and accept signals in the IPS, ACC and mPFC during internal exploration, external exploration and accept decisions. Statistics for this figure are reported with raw *p* values for descriptive illustration of the time courses. **(a).** In the IPS, the internal exploration signal remained significant in all three types of decision (top panel; internal exploration: $t_{23} = 2.253$, $p = 0.034$; middle panel, external exploration: $t_{23} = 2.285$, $p = 0.032$; bottom panel, accept: $t_{23} = 2.872$, $p = 0.009$). The IPS showed internal exploration signals independent of the impending decision. Besides, when an accept decision was made subsequently, the IPS also showed a negative and then positive external exploration signal (bottom panel; negative: $t_{23} = -2.398$, $p = 0.025$; positive: $t_{23} = 2.320$, $p = 0.03$) and a negative accept signal ($t_{23} = -3.016$, $p = 0.006$). In our task, higher external exploration or accept value indicates that additional internal exploration decision offers limited information gain, and that could be related to the decrease in IPS activity. **(b).** The ACC showed a negative internal exploration signal ($t_{23} = -2.592$, $p = 0.016$), suggesting that the ACC compares the relative advantage or

disadvantage of external exploration as opposed to the impending internal exploration. Critically, the ACC showed external exploration signal regardless of the type of impending decision, there was a positive external exploration signal when participants made external exploration (middle panel; peaked at 1.22s: $t_{23} = 2.094$, $p = 0.047$, peaked at 7.39s: $t_{23} = 2.280$, $p = 0.032$, peaked at 11.83s: $t_{23} = 3.374$, $p = 0.003$) or accept decisions (bottom panel; $t_{23} = 2.102$, $p = 0.047$). **Inset (top panel):** During internal exploration, participants often repeated selections of the same option. When all internal exploration decision in each repetition were analyzed, the external exploration signal was initially not significant ($t_{23} = 1.555$, $p = 0.134$), after the first decision in each repetition was removed, and only the second and later decisions were analysed, the external exploration signal became significant (top panel; $t_{23} = 3.149$, $p = 0.004$). **(c).** The mPFC showed general decision signal. During internal exploration, there were positive accept signals (peaked at -0.43s: $t_{23} = 2.474$, $p = 0.021$; peaked at 3.51s: $t_{23} = 2.838$, $p = 0.009$), after that, the mPFC switched to encode an internal exploration signal (top panel; $t_{23} = 2.666$, $p = 0.014$). In our task, options worth internal exploration are typically high in both uncertainty and average value. This pattern suggests that such options are often evaluated as accept decisions at first, but consideration of their uncertainty leads participants to withhold accept decisions and switch to internal exploration. During external exploration, the mPFC showed external exploration signal (middle panel; peaked at 7.65s: $t_{23} = 2.872$, $p = 0.009$, peaked at 11.85s: $t_{23} = 3.189$, $p = 0.004$), there were also negative internal exploration signals (peaked at -1.05s: $t_{23} = -2.536$, $p = 0.018$; peaked at 3.82s: $t_{23} = -2.671$, $p = 0.014$; peaked at 8.04s: $t_{23} = -2.684$, $p = 0.013$). During accept decisions, the mPFC reflected a positive accept signal (bottom panel; $t_{23} = 4.205$, $p < 0.001$). Besides, there was a negative internal exploration signal ($t_{23} = -3.373$, $p = 0.003$), consistent with a value-difference code in which mPFC emphasises the value of the current decision (accept) relative to its alternative (internal exploration). Again, these were consistent with a value difference code in which the mPFC emphasizes the value of the current decision (external exploration) compared with its alternative (internal exploration). Time-locked to the stimulus onset (solid black line). A dotted vertical line indicates the average reaction time (RT) for each decision type shown: Internal exploration (M = 3.76 s, SD = 1.56 s), external exploration (M = 2.75 s, SD = 1.19 s), and Accept (M = 3.06 s, SD = 1.50 s). * denotes $p < 0.05$, ** denotes $p < 0.01$, *** denotes $p \leqslant 0.001$. Shaded areas represent ± SEM.

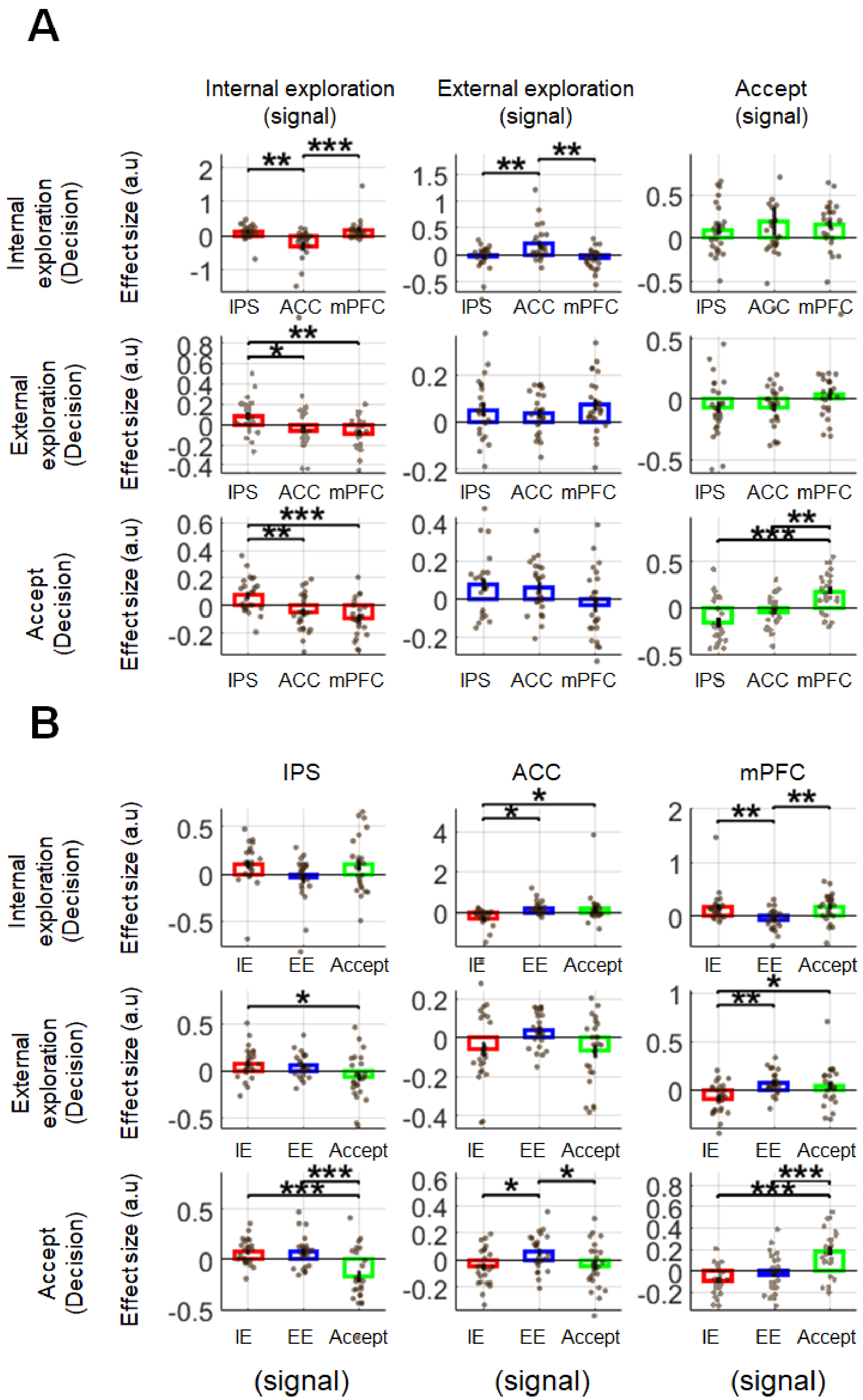
A
Internal exploration (signal)
External exploration (signal)
Accept (signal)
Internal exploration (Decision)
External exploration (Decision)
Accept (Decision)
Effect size (a.u)
IPS ACC mPFC
B
IPS
ACC
mPFC
IE EE Accept
(signal)

Supplementary Figure S9. Post-hoc contrasts by Region × Decision × Signal (FDR corrected) (n = 24). (A). We first examined the internal exploration signal in IPS (left panel). During internal exploration decisions, the internal exploration signal was greater in IPS than ACC (IPS: *Mean* = 0.103, *SD* = 0.225; ACC: *Mean* = −0.316, *SD* = 0.597; post-hoc FDR-corrected p = 0.001). During external exploration decisions, the internal exploration signal was greater in IPS (*Mean* = 0.080, *SD* = 0.171) than ACC (*Mean* = −0.058, *SD* = 0.178, p = 0.014) and mPFC (*Mean* = −0.089, *SD* = 0.162, p = 0.045). During accept decisions, the internal exploration signal was greater in IPS (*Mean* = 0.075, *SD* = 0.128) than ACC (*Mean* = −0.050, *SD* = 0.138, p = 0.036) and mPFC (*Mean* = −0.091, *SD* = 0.133, p = 0.002). Second, we examined the external exploration signal with a focus on ACC (middle panel). During internal exploration decisions, the external exploration signal was greater in ACC (*Mean* = 0.209, *SD* = 0.327) than IPS (*Mean* = −0.044, *SD* = 0.244, p = 0.003) and mPFC (*Mean* = −0.070, *SD* = 0.196, p = 0.002). (B). Third, we examined the signals in mPFC (right panel). During internal exploration decisions, the internal exploration signal (*Mean* = 0.166, *SD* = 0.305) was greater than the external exploration signal (*Mean* = −0.070, *SD* = 0.196; p = 0.008). During external exploration decisions, the external exploration signal (*Mean* = 0.071, *SD* = 0.121) was greater than the internal exploration signal (IE: *Mean* = −0.089, *SD* = 0.162; p = 0.007). During accept decisions, the accept signal (*Mean* = 0.179, *SD* = 0.213) was greater than the internal exploration signal (*Mean* = −0.091, *SD* = 0.133; $p < 0.001$) and the external exploration signal (EE: *Mean* = -0.033, *SD* = 0.181; $p < 0.001$). Taken together, these post-hoc contrasts strengthen the role in exploratory decisions across regions. IPS generally encodes the value of internal exploration regardless the nature of the decisions, while ACC encodes the value of external exploration, and mPFC shows decision general coding. * denotes $p < 0.05$; ** denotes $p < 0.01$; *** denotes $p \leq 0.001$. Error bars represent ± SEM.

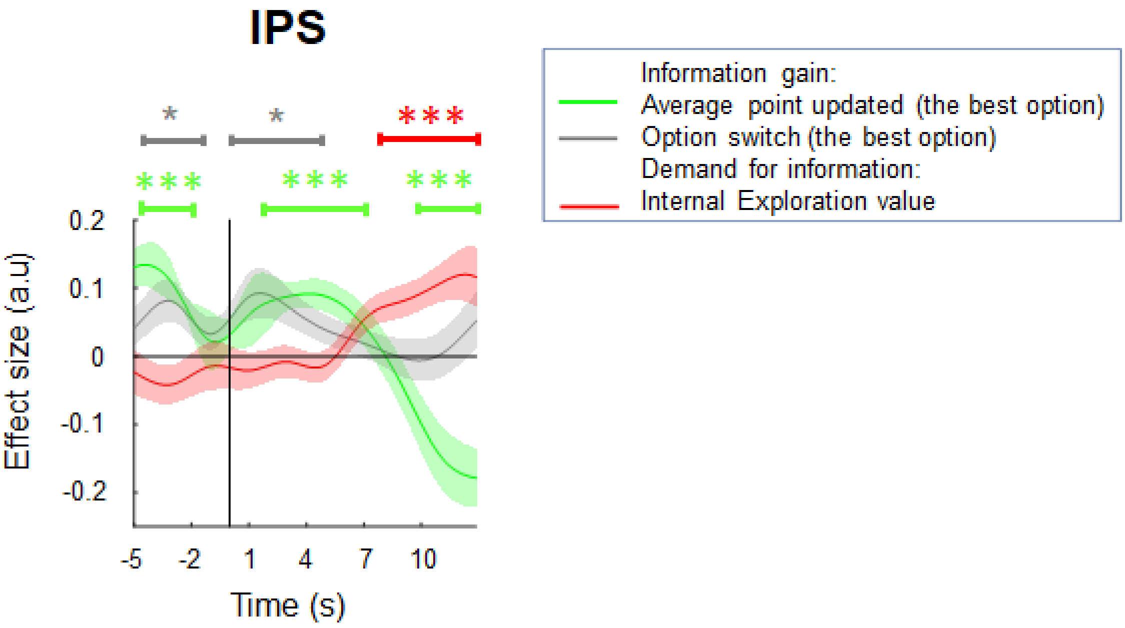

Supplementary Figure S10. Information gain and the demand for information signals in the IPS. Time courses of information gain and the demand for information. We defined the information gain signals as the updates of the average point of the best option and the switch to the best option. These two regressors were included after considering two major initiatives of making an internal exploration decision. First, the purpose of making an internal exploration

was to update an option by removing an unwanted dial (a smaller possible point gain) such that the option would be more appealing. Second, the individual may switch to a new best option if the average point of the internal explored option became the greatest after internal exploration, or may choose to retain the same best option. The analysis indicated two positive signals about the average point updated of the best option (peaked at -3.87s: t_{23} = 3.712, p = 0.001, peaked at 4.52s: t_{23} = 3.949, p = 0.001), and two positive signals about the best option switched (peaked at -2.82s: t_{23} = 2.613, p = 0.016, peaked at 2.22s: t_{23} = 2.567, p = 0.017). Further, the demand for information was defined by the internal exploration value. The analysis indicated a positive internal exploration signal (peaked at 10.77s: t_{23} =3.728, p = 0.001) and a negative signal about the best option's average point updated (peaked at 12.14s: t_{23} =3.728 = -3.655, p = 0.001). Leave-one-out peak selection for all time course analyses. Time-locked to the stimulus onset (solid black line). * denotes $p < 0.05$, ** denotes $p < 0.01$, *** denotes $p \leq 0.001$. Error bars represent ± SEM. Only the trials after internal exploration decision were included.

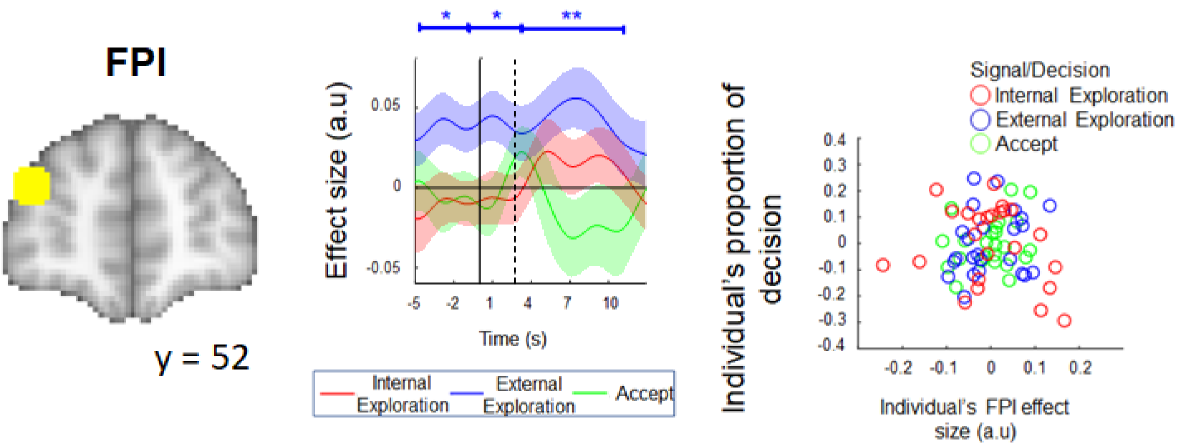

Supplementary Figure S11. Signals in the FPl. The coordinates were taken from Law et al. (2023)[4] (left panel). A time course analysis showing a significant signal in the FPl related to external exploration (middle panel; peaks observed at -3.06s: t_{23} = 2.483, p = 0.021; 1.53s: t_{23} = 2.703, p = 0.013; and 7.14s: t_{23} = 2.894, p = 0.008), but it was unrelated to internal exploration and accept values. There was no significant correlation between the peak signal of each participant and their proportions of internal exploration, external exploration, and accept decision made (right panel). Time-locked to the stimulus onset (solid black line). A dotted vertical line marks the average reaction time (RT) for external exploration (M = 2.75 s, SD = 1.19 s). * denotes $p < 0.05$; ** denotes $p < 0.01$. Shaded areas represent ± SEM.

Supplementary Table

Supplementary Table S1 – Model fit in BIC and Log-likelihood

Internal exploration value (type 1)

External exploration value (type)	Accept value (type)							
	BIC – Mean (SD)				Log-likelihood – Mean (SD)			
	1	2	3	4	1	2	3	4
1	459.662	618.582	605.671	569.832	-216.447	-295.907	-289.452	-271.532
	(166.695)	(211.710)	(212.056)	(172.080)	(83.158)	(105.733)	(105.817)	(85.905)
2	516.638	490.787	478.409	453.255	-244.935	-232.010	-225.821	-213.244
	(188.894)	(185.500)	(176.202)	(153.666)	(94.212)	(92.685)	(88.001)	(76.702)
3	498.729	451.874	442.471	418.800	-235.981	-212.553	-207.852	-222.279
	(174.845)	(154.413)	(145.270)	(126.043)	(87.228)	(77.171)	(72.568)	(87.177)
4	471.325	498.220	490.181	455.644	-222.279	-235.726	-231.707	-214.438
	(174.693)	(181.218)	(180.177)	(144.362)	(87.177)	(90.512)	(89.903)	(72.049)
5	459.662	438.442	434.657	397.573	-216.447	-205.837	-203.945	-185.403
	(166.695)	(141.991)	(137.139)	(105.902)	(13.158)	(70.977)	(68.447)	(52.927)

Internal exploration value (type 2)

External exploration value (type)	Accept value (type)							
	BIC – Mean (SD)				Log-likelihood – Mean (SD)			
	1	2	3	4	1	2	3	4
1	448.731	647.370	663.797	566.707	-210.982	-310.301	-318.515	-269.970
	(165.276)	(243.931)	(253.903)	(172.783)	(82.473)	(121.759)	(126.668)	(94.709)
2	510.222	517.826	525.274	454.319	-241.728	-245.529	-249.254	-213.776
	(189.860)	(205.147)	(206.489)	(153.095)	(94.708)	(102.411)	(103.027)	(76.422)
3	494.637	474.250	481.174	421.210	-233.935	-223.741	-227.203	-197.221
	(175.907)	(165.097)	(163.071)	(125.839)	(87.762)	(82.414)	(81.333)	(62.816)
4	461.594	519.604	529.567	453.124	-217.414	-246.418	-251.400	-213.178
	(174.728)	(201.600)	(205.753)	(143.826)	(87.205)	(100.612)	(102.628)	(71.806)
5	448.731	454.211	463.788	397.212	-210.982	-213.722	-218.511	-185.223
	(165.276)	(150.244)	(148.809)	(106.329)	(82.473)	(75.018)	(74.210)	(53.169)

Internal exploration value (type 3)

External exploration value (type)	Accept value (type)							
	BIC – Mean (SD)				Log-likelihood – Mean (SD)			
	1	2	3	4	1	2	3	4
1	385.347	586.623	572.421	560.925	-179.290	-279.928	-272.827	-267.079
	(123.224)	(184.131)	(185.298)	(167.231)	(61.616)	(92.008)	(92.555)	(83.530)
2	439.178	463.989	463.980	445.129	-206.206	-218.611	-218.606	-209.181
	(146.272)	(167.175)	(166.018)	(150.499)	(73.065)	(83.534)	(82.939)	(75.160)
3	427.210	424.416	425.476	410.223	-200.221	-198.824	-199.354	-191.728
	(136.486)	(136.249)	(135.469)	(123.933)	(68.209)	(68.109)	(67.704)	(61.911)
4	397.560	466.384	457.410	444.467	-185.396	-219.809	-215.322	-208.850
	(134.170)	(151.914)	(150.355)	(136.413)	(67.076)	(75.936)	(65.139)	(68.140)
5	385.347	404.158	401.566	388.064	-179.290	-188.696	-187.399	-180.648
	(123.224)	(114.831)	(113.545)	(101.673)	(61.616)	(57.507)	(56.824)	(50.876)

Internal exploration value (type 4)

**External exploration value (type)**	**Accept value (type)**							
	BIC – Mean (SD)				Log-likelihood – Mean (SD)			
	1	2	3	4	1	2	3	4
1	369.365	563.306	541.801	497.177	-171.299	-268.270	-257.517	-235.205
	(110.240)	(165.495)	(167.449)	(155.561)	(55.154)	(82.741)	(86.680)	(77.773)
2	427.423	454.912	454.813	447.461	-200.328	-214.073	-214.023	-210.347
	(137.051)	(156.952)	(156.219)	(148.116)	(68.436)	(78.387)	(78.013)	(73.968)
3	416.920	416.975	418.416	414.654	-195.076	-195.104	-195.824	-193.944
	(129.938)	(130.487)	(130.733)	(123.539)	(64.916)	(65.189)	(65.305)	(61.692)
4	383.047	454.567	441.716	418.302	-178.140	-213.900	-207.474	-195.767
	(124.311)	(140.884)	(139.199)	(130.572)	(62.148)	(70.469)	(69.610)	(65.264)
5	369.365	393.318	388.396	377.919	-171.299	-183.275	-180.814	-175.576
	(110.240)	(106.938)	(106.112)	(102.326)	(55.154)	(53.611)	(53.167)	(51.201)

Internal exploration value (type 5)

**External exploration value (type)**	**Accept value (type)**							
	BIC – Mean (SD)				Log-likelihood – Mean (SD)			
	1	2	3	4	1	2	3	4
1	361.257	548.604	522.921	477.867	-167.245	-260.919	-248.077	-225.550
	(105.013)	(156.107)	(159.884)	(148.357)	(52.537)	(78.060)	(79.884)	(74.076)
2	417.279	457.209	455.005	444.104	-195.256	-215.221	-214.119	-208.668
	(129.080)	(151.816)	(150.254)	(145.152)	(64.450)	(75.806)	(75.021)	(72.449)
3	406.971	414.234	414.596	409.015	-190.102	-193.734	-193.914	-191.124
	(122.403)	(127.378)	(126.994)	(119.598)	(61.148)	(63.612)	(63.418)	(59.696)
4	374.206	454.897	438.342	409.021	-173.719	-214.065	-205.787	-191.127
	(117.786)	(141.983)	(138.963)	(128.375)	(58.884)	(71.011)	(69.474)	(64.105)
5	361.257	397.480	388.639	375.377	-167.245	-185.357	-180.936	-174.305
	(105.013)	(110.894)	(108.673)	(103.269)	(52.537)	(55.551)	(54.419)	(151.62)

Internal exploration value (type 6)

**External exploration value (type)**	**Accept value (type)**							
	BIC – Mean (SD)				Log-likelihood – Mean (SD)			
	1	2	3	4	1	2	3	4
1	370.055	556.119	530.452	493.283	-171.644	-264.676	-251.842	-233.258
	(112.401)	(162.204)	(165.195)	(154.203)	(56.238)	(81.116)	(82.546)	(77.013)
2	421.947	463.749	460.890	444.401	-197.590	-218.491	-217.061	-208.817
	(133.210)	(156.479)	(154.319)	(145.822)	(66.537)	(78.149)	(77.061)	(72.793)
3	411.349	419.220	419.042	407.035	-192.291	-196.226	-196.137	-190.134
	(125.098)	(132.693)	(130.918)	(118.731)	(62.527)	(66.300)	(65.409)	(59.298)
4	381.317	461.956	445.253	416.392	-177.275	-217.594	-209.243	-194.812
	(123.631)	(148.307)	(144.228)	(130.235)	(61.822)	(74.169)	(72.102)	(65.035)
5	370.055	405.821	395.908	378.552	-171.644	-189.527	-184.570	-175.893
	(112.401)	(118.412)	(114.466)	(103.144)	(56.238)	(59.306)	(57.316)	(51.579)

**Supplementary Table S2<br>Identified regions from whole brain analyses for regressors-of-interest**					
**Internal exploration value**					
**Brodmann's areas**	**Brain region***	**MNI coordinates**	**Max Z-score**	**P-value**	**# voxels**
40	Intraparietal sulcus				
	- Left	(-10 -68 58)	5.16	4.72E-24	2315
6	Superior frontal gyrus				
	- Right	(22 14 56)	4.89	1.40E-12	913
	- Left	(-16 16 56)	4.48	8.34E-07	391
18	Lateral occipital cortex				
	- Left	(-32 -58 -30)	5.16	1.19E-07	466
37	Fusiform gyrus				
	- Right	(40 -64 -24)	4.25	6.56E-06	323
9	Superior frontal cortex				
	- Left	(-44 36 32)	4.74	4.54E-05	264
NA	Caudate				
	- Left	(-14 22 2)	4.19	0.000304	210
10	Frontal pole				
	- Left	(-30 68 4)	4.39	0.0258	101
39	Angular gyrus				
	- Right	(50 -46 34)	4.9	0.0283	99
**External exploration value**					
**Brodmann's areas**	**Brain region***	**MNI coordinates**	**Max Z-score**	**P-value**	**# voxels**
32	Anterior cingulate cortex	(0 -2 42)	4.54	1.16E-05	300
18	Secondary visual cortex				
	- Left	(-8 -92 24)	5.59	6.82E-39	4588
30	Posterior cingulate gyrus				
	- Left	(-6 -48 8)	4.59	6.68E-06	317
7	Visual motor cortex				
	- Right	(8 -54 66)	4.52	4.02E-05	263
	- Left	(-22 -58 64)	4.01	0.0469	87
6	Superior frontal gyrus				
	- Right	(24 0 58)	4.49	9.32E-05	239
	- Left	(-22 -6 54)	4.17	0.00291	149
	- Right	(32 -22 72)	4.26	0.00742	127
NA	Amygdala				
	- Left	(-24 -6 -18)	3.74	0.0405	90
**Accept value**					
**Brodmann's areas**	**Brain region***	**MNI coordinates**	**Max Z-score**	**P-value**	**# voxels**

32	Medial prefrontal cortex	(-6 48 -2)	3.96	1.19E-07	448
18	Secondary visual cortex				
	- Left	(-24 -94 -10)	4.84	2.38E-06	350
39	Angular gyrus				
	- Left	(-52 -62 38)	4.33	0.00633	131
24	Ventral anterior cingulate				
	- Left	(-4 -8 50)	4	0.0168	109
NA	Caudate				
	- Left	(-10 12 -10)	4.39	0.041	90
	- Right	(10 12 -6)	4.53	0.0497	86

Supplementary Information references

1. Behrens, T. E. J., Woolrich, M. W., Walton, M. E. & Rushworth, M. F. S. Learning the value of information in an uncertain world. *Nat Neurosci* 10, 1214–1221 (2007).

2. Quilodran, R., Rothé, M. & Procyk, E. Behavioral Shifts and Action Valuation in the Anterior Cingulate Cortex. *Neuron* 57, 314–325 (2008).

3. Wessel, J. R., Danielmeier, C., Bruce Morton, J. & Ullsperger, M. Surprise and error: Common neuronal architecture for the processing of errors and novelty. *Journal of Neuroscience* 32, 7528–7537 (2012).

4. Law, C. K., Kolling, N., Chan, C. C. H. & Chau, B. K. H. Frontopolar cortex represents complex features and decision value during choice between environments. *Cell Rep* 42, (2023).
