# Supplementary material for: Distinct contributions of prefrontal, parietal, and cingulate signals to exploratory decisions
Source: Commun Biol. 2026 Jan 22;9:272. doi: 10.1038/s42003-026-09550-w (PMC12916896; doi:10.1038/s42003-026-09550-w)
Supplement: Supplementary file 3 — Reporting-summary [file 42003_2026_9550_MOESM3_ESM.pdf]

nature portfolio

Corresponding author(s): Victor K.S. Chan
23039036r@connect.polyu.hk

Last updated by author(s): Dec 22, 2025

Reporting Summary

Nature Portfolio wishes to improve the reproducibility of the work that we publish. This form provides structure for consistency and transparency in reporting. For further information on Nature Portfolio policies, see our Editorial Policies and the Editorial Policy Checklist.

Statistics

For all statistical analyses, confirm that the following items are present in the figure legend, table legend, main text, or Methods section.

n/a	Confirmed	
☐	☒	The exact sample size (*n*) for each experimental group/condition, given as a discrete number and unit of measurement
☐	☒	A statement on whether measurements were taken from distinct samples or whether the same sample was measured repeatedly
☐	☒	The statistical test(s) used AND whether they are one- or two-sided<br>*Only common tests should be described solely by name; describe more complex techniques in the Methods section.*
☒	☐	A description of all covariates tested
☒	☐	A description of any assumptions or corrections, such as tests of normality and adjustment for multiple comparisons
☐	☒	A full description of the statistical parameters including central tendency (e.g. means) or other basic estimates (e.g. regression coefficient) AND variation (e.g. standard deviation) or associated estimates of uncertainty (e.g. confidence intervals)
☐	☒	For null hypothesis testing, the test statistic (e.g. *F*, *t*, *r*) with confidence intervals, effect sizes, degrees of freedom and *P* value noted<br>*Give P values as exact values whenever suitable.*
☒	☐	For Bayesian analysis, information on the choice of priors and Markov chain Monte Carlo settings
☒	☐	For hierarchical and complex designs, identification of the appropriate level for tests and full reporting of outcomes
☐	☒	Estimates of effect sizes (e.g. Cohen's *d*, Pearson's *r*), indicating how they were calculated

Our web collection on statistics for biologists contains articles on many of the points above.

Software and code

Policy information about availability of computer code

Data collection	Data collection was done using Psychtoolbox (v 3.0.16) http://psychtoolbox.org/, in National Institute of Information and Communications Technology (CiNet, NICT, Osaka, Japan). Neuroimaging data were acquired using a 3 Tesla Siemens Magnetom Prisma MR scanner at Center for Information and Neural Networks and a 32-channel phase array coil.
Data analysis	Data analysis was done using Matlab 2022b https://www.mathworks.com/products/matlab.html, custom MATLAB code can be found at https://osf.io/zcfxy (https://doi.org/10.17605/OSF.IO/ZCFXY), and FSL (FMRIB Software Library v6.0.5) https://fsl.fmrib.ox.ac.uk/fsl/docs/;

For manuscripts utilizing custom algorithms or software that are central to the research but not yet described in published literature, software must be made available to editors and reviewers. We strongly encourage code deposition in a community repository (e.g. GitHub). See the Nature Portfolio guidelines for submitting code & software for further information.

Data

Policy information about availability of data

All manuscripts must include a data availability statement. This statement should provide the following information, where applicable:

- Accession codes, unique identifiers, or web links for publicly available datasets
- A description of any restrictions on data availability
- For clinical datasets or third party data, please ensure that the statement adheres to our policy

The data and code supporting the findings in this study can be found at https://osf.io/zcfxy (https://doi.org/10.17605/OSF.IO/ZCFXY).

Research involving human participants, their data, or biological material

Policy information about studies with human participants or human data. See also policy information about sex, gender (identity/presentation), and sexual orientation and race, ethnicity and racism.

Reporting on sex and gender	13 female and 10 male subjects were recruited, gender was determined by self-reporting. One subject did not report. No variables about sex and gender were used in the current study.
Reporting on race, ethnicity, or other socially relevant groupings	No variables about race, ethnicity, or other socially relevant groupings were used in the current study.
Population characteristics	No variables about population characteristics were used in the current study.
Recruitment	26 subjects were recruited in National Institute of Information and Communications Technology (CiNet, NICT, Osaka, Japan) by convenience sampling, 2 were excluded from data analysis due to excessive movement inside fMRI scannings.
Ethics oversight	Protocol was approved by the ethics committees of The Hong Kong Polytechnic University and National Institute of Information and Communications Technology, Japan, and methods conformed to the relevant guidelines and regulations.

Note that full information on the approval of the study protocol must also be provided in the manuscript.

Field-specific reporting

Please select the one below that is the best fit for your research. If you are not sure, read the appropriate sections before making your selection.

☒ Life sciences ☐ Behavioural & social sciences ☐ Ecological, evolutionary & environmental sciences

For a reference copy of the document with all sections, see nature.com/documents/nr-reporting-summary-flat.pdf

Life sciences study design

All studies must disclose on these points even when the disclosure is negative.

Sample size	26 healthy human subjects were involved in the experiment.
Data exclusions	2 subjects were excluded from data analysis due to excessive movement during MRI scanning.
Replication	For the Region-of-interest (ROI) analysis, a leave-one-subject-out procedure was repeated 23 times, and ultimately averaged.
Randomization	All subjects were used for experiements. The types of trials were randomly determined by a computer program.
Blinding	Data analysis was done regardless of the identity of the subjects. During the decision-making task, each subject was told that the reward of each trial was randomized.

Reporting for specific materials, systems and methods

We require information from authors about some types of materials, experimental systems and methods used in many studies. Here, indicate whether each material, system or method listed is relevant to your study. If you are not sure if a list item applies to your research, read the appropriate section before selecting a response.

Materials & experimental systems

n/a	Involved in the study	
☒	☐	Antibodies
☒	☐	Eukaryotic cell lines
☒	☐	Palaeontology and archaeology
☒	☐	Animals and other organisms
☒	☐	Clinical data
☒	☐	Dual use research of concern
☒	☐	Plants

Methods

n/a	Involved in the study	
☒	☐	ChIP-seq
☒	☐	Flow cytometry
☐	☒	MRI-based neuroimaging

Plants

Seed stocks

N/A.

Novel plant genotypes

N/A.

Authentication

N/A.

Magnetic resonance imaging

Experimental design

Design type

Event related fMRI.

Design specifications

Each subject performed 100 trials. The details of each trial are summarized in Fig. 1A. Each trial started with a fixation cross with an inter-trial interval (ITI) ranging between 3 and 6 seconds. In each trial, subjects were presented with an option in one of nine potential positions, represented by black boxes (Fig. 1A). Each option consisted of four dials, with the colored portions indicating the range of possible points that could be earned. Unbeknown to subjects, only one dial identity was related to the actual points earned by each option, such that options containing more variable dials had greater uncertainties. Subjects were required to choose between three possible actions: (1) Accept the current option to end a trial and earn the associated points, which also lead to an outcome phase showing the actual points earned, ranging between 1 and 3 seconds); (2) Perform internal exploration by randomly eliminating one dial from the selected option to decrease its uncertainty (interstimulus interval ranged between 1 and 3 seconds), with a maximum of three times per option; or (3) Pursue external exploration by unveiling a new option from the remaining black boxes (interstimulus interval ranged between 1 and 3 seconds), limited to eight explorations per trial. Each internal or external would cost one point. To help decision-making, particularly for external exploration, the average point value of all options including both revealed and hidden ones within the trial would be displayed at the top right corner.

Behavioral performance measures

We developed a General Linear Model (GLM) to describe subjects' decisions.

Acquisition

Imaging type(s)

Functional

Field strength

3 Tesla

Sequence & imaging parameters

The details of the sequence and imaging parameters are reported in the Methods. fMRI data were acquired using a 3 Tesla Siemens Magnetom Prisma MR scanner and a 32-channel phase array coil. Echo-planar imaging data was obtained from 72 slices [whole brain coverage, repetition time (TR) = 2000 ms, echo time (TE) = 30 ms, field of view (FOV) = 200 x 200, flip angle = 75°. Field maps were acquired to correct for signal distortions using a dual echo 2D gradient echo sequence (TR = 75 ms, TE1 = 5.16 ms, TE2 = 7.62 ms, FOV = 200 x 200, flip angle = 90 deg, 2.5 x 2.5 x 2.5 mm3 resolution). For each subject a high-resolution T1 image was acquired using a 1mm3 anatomical scan (208 slices, TR = 1900 ms, TE = 2.48 ms, FOV = 256 x 256, flip angle = 9°) for accurate co-registration of fMRI images to individual anatomy space and for reconstructing cortical surfaces.

Area of acquisition

Whole brain

Diffusion MRI ☐ Used ☒ Not used

Preprocessing

Preprocessing software

The preprocessing of the fMRI data used FMRIB's Software Library (FSL)(Ratcliff & Smith, 2004).

Normalization

Functional images were aligned with each subject's anatomical scan and transformed to Montreal Neurological Institute (MNI) space (Jenkinson & Smith, 2001).

Normalization template

We used the standard MNI space.

Noise and artifact removal

The fMRI data were preprocessed using brain extraction (Brain Extraction Tool)(Smith, 2002), motion correction (FMRIB's Linear Image Registration Tool)(Beckmann et al., 2003), Gaussian spatial smoothing with full width at half maximum (FWHM) sizes of 5 mm, field-map correction for distorted signal(Beckmann et al., 2003), and high-pass temporal filtering (3 dB cut-off of 100s).

Volume censoring | No volume censoring was used.

Statistical modeling & inference

Model type and settings

The whole-brain analysis was done using a univariate GLM approach. We entered the internal exploration value, external exploration value, accept value, cumulative cost that were time-locked to stimulus onset as regressors in the GLM, convolved with a canonical hemodynamic function (two-gamma model)(Glover, 1999) to provide idealised hemodynamic responses. 12 additional nuisance regressors were also included. Four parametric regressors related to the number of existing options, the accumulative gain across trial, the trial gain and the sum of the points of all existing options, time-locked to the stimulus onset, were included. Six box car regressors related to subjects' motor movements, time-locked to their motor movements; interstimulus interval (isi), time-locked to stimulus onset, intertrial interval (iti), time-locked to trial onset; outcome phase, time-locked to outcome phase onset; two types of event constants (duration = 1 and from trial onset to every decision), time-locked to subjects' decisions, were included. Two regressors related to the average BOLD signal in the cerebrospinal fluid (CSF) and white matter (WM) were included. At group level, FMRIB's local analysis of mixed effects was applied with outlier deweighing. All images were cluster-corrected results with voxel inclusion threshold of z = 3.1, and threshold of cluster significance at 0.05 (p-value).

For the Region-of-interest (ROI) analysis, the extracted ROI time courses were time-locked to stimulus onset. For each subject, the ROI activities at each time point were regressed via a GLM, such that time courses of beta weight for each regressor in the GLM were acquired. The beta weight time courses were then group averaged. The size of each peak was extracted for each subject, followed by running a one-sample t test to test whether each peak in the group time courses significantly differed from zero. A leave-one-subject-out procedure was repeated 23 times, and ultimately averaged, to find the peak time (within a window of -5 to 13s). Peak window selection was done by finding the full-width half-maximum of the peak in the group time course, which was determined as the period between two time-points at the peak half maximum value (i.e., regression weight).

Effect(s) tested | The full models are described in the methods including all regressors entered in the analysis. See GLM1.

Specify type of analysis: ☐ Whole brain ☐ ROI-based ☒ Both

Anatomical location(s) | We extracted the time series of brain regions previously identified in the whole brain analysis (i.e., IPS, ACC, mPFC). A mask was created to extract the activity of the IPS by centering a sphere of 3mm radius at the coordinates taken from Mars et al.(Mars et al., 2011). The process was repeated for both ACC and mPFC, referencing coordinates from Kolling et al.(Kolling et al., 2012) and Blair et al.(Blair et al., 2006) respectively.

Statistic type for inference (See Eklund et al. 2016) | Cluster inference using a cluster-defining threshold with |Z| > 3.1.

Correction | FWE-corrected threshold of p = 0.001.

Models & analysis

n/a	Involved in the study
☒	☐ Functional and/or effective connectivity
☒	☐ Graph analysis
☒	☐ Multivariate modeling or predictive analysis
